# Evaluation and Validation of Reference Genes for Normalization of Quantitative Real-Time PCR Based Gene Expression Studies in Peanut

**DOI:** 10.1371/journal.pone.0078555

**Published:** 2013-10-22

**Authors:** Dumbala Srinivas Reddy, Pooja Bhatnagar-Mathur, Katamreddy Sri Cindhuri, Kiran K. Sharma

**Affiliations:** International Crops Research Institute for the Semi-Arid Tropics (ICRISAT), Patancheru, Andhra Pradesh, India; National Institute of Environmental and Health Sciences, United States of America

## Abstract

The quantitative real-time PCR (qPCR) based techniques have become essential for gene expression studies and high-throughput molecular characterization of transgenic events. Normalizing to reference gene in relative quantification make results from qPCR more reliable when compared to absolute quantification, but requires robust reference genes. Since, ideal reference gene should be species specific, no single internal control gene is universal for use as a reference gene across various plant developmental stages and diverse growth conditions. Here, we present validation studies of multiple stably expressed reference genes in cultivated peanut with minimal variations in temporal and spatial expression when subjected to various biotic and abiotic stresses. Stability in the expression of eight candidate reference genes including *ADH3*, *ACT11*, *ATPsyn*, *CYP2*, *ELF1B*, *G6PD*, *LEC* and *UBC1* was compared in diverse peanut plant samples. The samples were categorized into distinct experimental sets to check the suitability of candidate genes for accurate and reliable normalization of gene expression using qPCR. Stability in expression of the references genes in eight sets of samples was determined by geNorm and NormFinder methods. While three candidate reference genes including *ADH3*, *G6PD* and *ELF1B* were identified to be stably expressed across experiments, *LEC* was observed to be the least stable, and hence must be avoided for gene expression studies in peanut. Inclusion of the former two genes gave sufficiently reliable results; nonetheless, the addition of the third reference gene *ELF1B* may be potentially better in a diverse set of tissue samples of peanut.

## Introduction

Gene expression studies have become increasingly important to understand the molecular mechanisms in animal, human, microorganism, and plant systems [1-4]. Gene expression levels have been determined by techniques including Northern blotting, RNase protection assay, semi-quantitative reverse-transcription PCR, and quantitative real-time PCR (qPCR) [4]. However, qPCR has gained importance over the rest owing to its high sensitivity, accuracy, speed, and high-throughput analysis. The main advantages with the qPCR analysis are ability to detect low-abundance mRNAs [5], quantify mRNA copy number [6], and need for relatively lower amount of test materials and no post-PCR gel analysis, etc. [7,8].

Nevertheless, a substantial technical variability associated with qPCR may exist due to inherent differences in samples, sample collection, quantity and quality of input RNA, reverse transcription and PCR efficiency, and pipetting errors [9]. In order to minimize these, the most common practice is to normalize the gene of interest with the reference gene (an internal control gene), which is also subjected to the similar errors in cDNA preparation, thereby, making results from qPCR more reliable than absolute quantification. The ideal reference gene should notably express stably across the developmental stages and under variable experimental conditions. However, selection of an unstable reference gene can add large unpredictable error to the analysis and result in incorrect evaluations [10]. Several studies have shown that no single internal control gene is universal for use as a reference gene for all experiments [11-13]. Different samples or treatments may require the re-evaluation of a suitable reference gene, since changing experimental conditions can sometimes cause a suitable reference gene to become unstable [14]. Hence, the reference genes that do not show variable expression levels in different cells and tissues under different conditions must be the choice so as to reduce measurement errors.

Cellular homeostasis genes, more commonly known as housekeeping genes that are involved in basic and ubiquitous cellular processes such as components of the cytoskeleton, glycolytic pathway, protein folding, protein degradation, and synthesis of ribosome subunits are mostly used as reference genes [9]. The most frequently used housekeeping genes that have been validated as suitable reference genes in many plants include β-actin (*ACT*), α-tubulin (*TUA*), ubiquitin (*UBQ*), glyceraldehde-3-phosphate dehydrogense (*GAPDH*), *18S* or *26S* ribosomal RNA and elongation factors (EF) etc. [15-19]. However, transcript levels of housekeeping genes too vary considerably across the developmental stages and under variable conditions [20], thereby, necessitating the selection of other multiple stably expressed reference genes to be considered for accurate normalization of gene expression studies [21]. Reference gene validation have been reported in a number of crop plants such as *Oryza sativa* L. [22], *Triticum aestivum* L. [23], *Zea mays* L. [24], *Solanum tuberosum* L. [15], *Solanum lycopersicum* L. [25], *Chrysanthemum* [19], *Vitis vinifera* L. [26], *Brassica rapa* L. [27], *Brassica napus* L. [28], *Brassica juncea* L. [9] *Arabidopsis* [12] *Glycine max* L. [16,29-33], and *Cicer arietinum* L. [17]. However, except for soybean and chickpea, very few studies have been conducted to validate reference genes for qPCR in legume crops, thus necessitating a need to validate experiment-specific reference genes from legumes, including peanut where a major emphasis is on the development of transgenic peanuts for various biotic and abiotic constraints and nutritional enhancement.

Peanut (*Arachis hypogaea* L.) is the second-most important grain legume crop cultivated in over 100 tropical and subtropical countries of the world [34] and is an important oilseed cash crop containing 48–50% oil and 20–25% protein. The major abiotic factors affecting peanut production include drought, high temperature, low soil fertility, low soil pH and iron chlorosis. Among the biotic factors, diseases caused by fungi, viruses, bacteria, nematodes, and foliar and soil insect pests significantly affect peanut productivity [35]. However, most of these agronomical traits are difficult to breed by conventional selection techniques due to little genetic variation within cultivated peanut. Modern biotechnology approaches including marker-assisted selection, tissue culture, embryo rescue and genetic transformation have been employed in crop improvement programs worldwide including peanut [36]. The use of transgenic technology potentially offers a targeted gene-based approach for the genetic enhancement of field crops. Moreover, with the advent of genetic transformation technology for crop improvement, molecular characterization of transgenic events need to be carried out at various stages from identification of the transgenic event to transgene integration, copy number detection and gene expression.

High-throughput molecular characterization of transgenic events is now possible with the introduction of qPCR based techniques. While, the qPCR based gene expression studies requires reference gene identification, only a limited number of reference gene validation studies have been carried out in peanut [37-39]. Hence, in recognition of the importance of reference genes for the normalization of qPCR data and a need to identify species-specific and experimental conditions-specific reference genes, the present study was conducted to validate suitable reference genes with minimal variations in temporal and spatial expression in cultivated peanut subjected to various biotic (fungal and viral) and abiotic (salt and drought) stresses. Here, we have selected eight candidate reference genes including alcohol dehydrogenase (*ADH3*), actin (*ACT11*), ATP synthase (*ATPsyn*), cyclophilin (*CYP2*), elongation factor 1B (*ELF1B*), glucose-6-phospahate 1-dehydrogenase (*G6PD*), lectin (*LEC*) and ubiquitin-conjugating enzyme (*UBC1*) through bibliographic reviews of studies in crop plants such as peanut, soybean and cotton followed by an *in silico* analysis. We have compared the expression stability of these candidate reference genes in diverse samples of peanut categorized into distinct experimental sets to check their suitability as stable reference genes for accurate and reliable normalization of gene expression using qPCR.

## Materials and Methods

### Plant Material

Peanut (*Arachis hypogaea* L.) varieties including JL24, TAG24, CS39, ICGV 86699, ICGV 06040, ICGV 91114, ICGV 00350 and ICGV 05155 were obtained from the Groundnut Breeding Unit of ICRISAT. Peanut plants were grown in 6 inch pots containing 3.5 kg of alfisol:sand:compost mixture (3:2:1; 20 % water holding capacity) under greenhouse conditions with 28/20 °C day/night temperature. Leaf samples were collected 3 days after shoot emergence (DAE) from all peanut varieties grown in the greenhouse. Different tissue samples from varieties JL24 and ICGV 86699 including leaf sample of early stage (LES), cotyledons, stems and roots were collected 3 days after shoot emergence (DAE), whereas leaf sample of flowering stage (LFS), immature pods and immature seeds were collected from plants at the pegging stage. The collected tissue samples were immediately frozen in liquid nitrogen and stored in -80 °C until RNA extraction. 

### Biotic and abiotic stress treatments

For stress treatments, 7 day-old seedlings of cultivars JL24 and ICGV 86699 were used for virus challenging experiments using the Tobacco streak virus (TSV) and Peanut bud necrosis virus (PBNV), while Late Leaf Spot (LLS) caused by *Phaeoisariopsis personata*, rust caused by *Puccinia arachidis* and abiotic stress treatments (salinity and drought) were imposed at early flowering stage. For TSV and PBNV infection, the virus inoculum was prepared from the infected plant leaf samples, (TSV from French bean (*Phaseolus vulgaris*) and PBNV from peanut) and infected to peanut plants by mechanical sap transmission method as described by Kumar and Waliyar [40]. Viral infection symptoms (necrotic lesions) were observed after 3-4 days of inoculation (DOI) on the inoculated mature leaves, and infection was confirmed by DAC-ELISA as described by Kumar and Waliyar [40]. Young leaf samples were collected in triplicates from the infected peanut plants after 5 DOI. 

Spores of LLS and rust were used for fungal infections. Spore collection, inoculum preparation, and inoculation methods for LLS and rust were conducted as described by Subrahmanyam et al. [41]. The spore suspension (30000 spores /mL) was sprayed onto peanut plants at early flowering stage, maintained under controlled conditions at 23 °C with 95 % relative humidity and 12 h photoperiod. Symptoms were evaluated 10 DOI, and leaf samples from control and inoculated peanut plants showing symptoms were collected separately.

Drought and salinity stress was imposed on peanut plants under greenhouse conditions. Plants were given regular irrigation before abiotic stress treatments and irrigated control plants were maintained as such. For drought stress, the water supply was withheld for 5 d followed by leaf sample collection, while the salinity stress was imposed by completely saturating the pots containing plants with 40 mM NaCl, followed by leaf sampling after 24 h.

### RNA isolation

Total RNA was extracted from peanut plants using 50 mg of tissue using NucleoSpin RNA plant kit (Macherey-Nagel, Duren, Germany) following the recommended procedures including in-column DNAse1 treatment. The isolated total RNA was tested for DNA contamination in PCR using *ELF1B* and *ADH3* primer pairs. The DNA contaminated samples were retreated with DNAseI (Macherey-Nagel, Duren, Germany) in tubes and re-purified using NucleoSpin RNA clean-up kit (Macherey-Nagel, Duren, Germany). The concentration and purity of RNA was determined using NanoVue plus spectrophotometer (GE health care, USA) and the absorbance at 260/280 nm ranging from 1.8 to 2.0 were selected for further analysis. Integrity of the RNA was further checked by electrophoresis through 1.4 % agarose gel. The total RNA isolated was diluted to 100 ng/µl concentration and aliquoted for use in PCR.

### Selection of reference genes and primer design

The candidate reference genes were selected through bibliographic reviews of studies in crop plants such as peanut (*LEC* [42]; *ADH3* [37]), soybean (*ATPsyn* [43]; *ACT11*, *ELF1B*, *CYP2* and *G6PD* [29]), and cotton (*UBC1*) [44]) followed by an *in silico* analysis using the BLAST tools of the NCBI database [45]. For instance, a previously selected EST in soybean was submitted to the BLASTN tool to obtain EST orthologous in peanut (Table 1). Subsequently, NCBI non-redundant protein sequence database (nr) was used to confirm the sequence function using BLASTX tool (http://blast.ncbi.nlm.nih.gov/Blast). Since the genome sequence of peanut is not known, alignments were made with relevant gene orthologous in *Arabidopsis* using BLASTN with optimization to ‘somewhat similar sequences’ before primer design to ensure the primer pairs span at least one intron. Primers were designed using primer analysis software PRIMER 3.0 (http://frodo.wi.mit.edu/primer3/) by considering the following parameters: (a) product size range: 100-160 bp; (b) primer size: 20-22 bp; (3) GC content 50 %. The EST GenBank accession number, primer sequence, amplicon length and primer locations are listed in Table 1.

**Table 1:** Details of the candidate reference genes of peanut and their primer sequences used for validation.

Gene	^a^Acc. no	^b^Gene function	^c^Primer sequence 5’ to 3’ (FP/RP)	Amplicon length (bp)		^f^Primers location	^g^Efficiency
				^d^ DNA	^e^cDNA		
*ACT11*	GO339334	Cytoskeletal structural protein	ATGCTAGTGGTCGTACAACTGG	400	108	D	0.99
			CTAGACGAAGGATAGCATGTGG				
*ADH3*	EG529529	Catalyzes the inter conversion of alcohols and aldehydes or ketones	GCTTCAAGAGCAGGTCACAAGT	450	143	D	1.00
			GAGACATCCTCCTTCGTGCATA				
*ATPsyn*	GO338918	ATP synthesis	AGGCAAACTTGCTCTCAGAGTC	450	151	D	1.03
			ATCATAGCCTCAGCGCCAAGAT				
*CYP2*	EE127717	Protein folding	GCTCCAAGTTTGCCGATGAGAA	161	161	S	1.00
			AACAACTTGGCCGAACACCA				
*ELF1B*	EE126175	Translational elongation	AAGCTTCCCTGGCAAAGCTCAA	650	153	D	0.99
			TTCCTCAGCTGCCTTCTTATCC				
*G6PD*	EG030635	Glucose-metabolic process	ACCATTCCAGAGGCTTATGAGC	500	151	D	1.00
			AAGGGAGTGACTTGAACTCTCC				
*LEC*	ES722311	Mannose/glucose binding	TCCAAGCACAGTTCAGCTTCGT	148	148	S	0.97
			TTCTGGGCAGTTTGAGGGTCAA				
*UBC1*	DQ887085	Ubiquitin- dependent Protein catabolic process	TTAAAGAGCAATGGAGCCCTGC	700	149	D	1.00
			ATACTTCTGTGTCCAGCTGCGT				
*PBNVnp^h^*	AY512652	Virus nucleocapsid protein	GGCTAGTATGGTTGAGAAGAGC	156	156	S	0.98
			AGAGGACCTCCAATACAGAGCA				
*AtDREB1A^h^*	AB007787	Transcription factor	AATCCCGGAATCAACTTGCGCT	134	134	S	1.00
			AAATAGCCTCCACCAACGTCTC				

a EST GenBank accession number.

b Gene description based on homology with Arabidopsis proteins.

c Forward (upper line) and reverse (lower line) primer sequences.

d Approximate length of the PCR amplified fragment with DNA template.

e Length of the PCR amplified fragment with cDNA template.

f Primers location on two exons (D), or on single exon (S).

g PCR efficiencies of primer pairs, measured using slandered curves.

h not reference genes, used for gene expression normalization.

### PCR analysis for specificity and efficiency of primers

In order to test the specificity of the primers and suitable reaction conditions, the primer sets were initially tested by standard PCR reaction with Mastercycler Gradient (Eppendorf, Germany) with temperature gradient (56 °C to 66 °C) using both DNA and cDNA templates. Genomic DNA was isolated from leaf samples of peanut variety JL24 using NucleoSpin plant II midi kit (Macherey-Nagel, Duren, Germany) following the manufacture’s protocol. The cDNA synthesis was carried out using the Thermoscript**^®^** RT-PCR system (Invitrogen-life technologies, USA) with total RNA samples according to the manufacturer’s instructions. Primer titrations were carried out to check the effect of primer concentrations in PCR. Template gradient PCR was carried out with different concentrations of cDNA to check the efficiency of the primers by constructing standard curves for each set of primers. All these qPCRs were carried out in Realplex (Eppendorf, Germany), Real Time PCR system using 2X SensiMix^TM^ SYBR No-ROX (Bioline, UK) kit and 400 nM of each primer was used in template gradient PCRs. The reaction conditions were set as 10 min at 95 °C (polymerase activation); 45 cycles of 15 s at 95 °C, 15 s at 62 °C with fluorescent signal recording and 15 s at 72 °C. At the end, a final step of 15 s at 95 °C, 30 s at 58 °C and fluorescence measurement at each 0.5 °C variation from 58 °C to 95 °C in 20 min was included to obtain the melting curve. For each sample, three technical replicates were performed and Cq values were taken for analysis after drift correction. All the PCR amplified products were verified by 2 % agarose gel electrophoresis with SYBR safe DNA gel stain (Invitrogen-life technologies, USA) prior to sequencing the amplified products to check the PCR product specificity.

### Real Time qPCR analysis

One step quantitative reverse transcriptase PCR (qRT-PCR) reactions were carried out in Realplex PCR system (Eppendorf, Germany) using 100 ng of total RNA, 2X SensiFAST^TM^ SYBR No-ROX one-Step kit (Bioline, UK), and 400 nM of each primer. The reaction conditions were set as: 10 min at 45 °C (Reverse transcription), 2 min at 95 °C (polymerase activation), 40 cycles of 10 s at 95 °C, and 20 s at 60 °C with fluorescent signal recording for amplification. At the end, a final step of 15 s at 95 °C, 30 s at 58 °C followed by fluorescence measurement at each 0.5 °C variation from 58 °C to 95 °C in 20 min was included to obtain the melting curve. Each sample was tested in three technical replicates.

### Data analysis

Expression levels of the eight candidate reference genes in all the sample pools were determined by the number of cycles needed for the amplification-related fluorescence to reach a specific threshold level of detection (quantification cycle Cq). The efficiency (E) of each primer pair was calculated based on slope of the line (E = 10^-1/slope^) considering an ideal value range of 0.95 to 1.0.

To carry out an in-depth data analysis, 31 diverse samples were categorized under eight experimental sets comprising of condition-specific samplings (Table 2). While the first experimental set included all 31 diverse peanut tissue samples, the second experimental set comprised of tissue samples of varieties JL24 and ICGV 86699 at vegetative stages [cotyledon, early young leaf (LES), stem and root], whereas the third set included tissue samples at reproductive stages (leaf, pods and immature seeds). The fourth set included total samples of both vegetative and reproductive stages. The peanut leaf samples from virus (TSV and PBNV) infected and uninfected controls were included in fifth experimental set, while the LLS and rust infected/non-infected leaf samples at flowering stage were included in sixth experimental set as foliar diseases. The seventh set comprised of leaf samples from salt and drought stressed treatments (abiotic stress), whereas the eighth experimental set included leaf samples from eight diverse peanut cultivars as described in the plant material section (Table 2).

**Table 2:** Details of the peanut tissue sample used for candidate reference genes validation.

S.No.	**Sample**	**Description of the tissue sample**	**Sample set no.**
1	Rt_JL24	Root tissue of JL24	1,2,4
2	St_JL24	Stem tissue of JL24	1,2,4
3	Ct_JL24	Cotyledon tissue of JL24	1,2,4
4	Rt_699	Root tissue of ICGV 86699	1,2,4
5	St_699	Stem tissue of ICGV 86699	1,2,4
6	Ct_699	Cotyledon tissue of ICGV 86699	1,2,4
7	IP_JL24	Immature pods tissue of JL24	1,3,4
8	IS_JL24	Immature seed tissue of JL24	1,3,4
9	LFS_JL24	Leaf tissue of JL24 variety at Flowering Stage	1,3,4
10	IP_699	Immature pods tissue of ICGV 86699	1,3,4
11	IS_699	Immature seed tissue of ICGV 86699	1,3,4
12	LFS_699	Leaf tissue of ICGV 86699 peanut at Flowering Stage	1,3,4,6,7
13	TSV_699	Leaf tissue of TSV infected ICGV 86699	1,5
14	TSV_JL24	Leaf tissue of TSV infected JL24	1,5
15	PBNV_JL24	Leaf tissue of PBNV infected JL24	1,5
16	LS1_699	Leaf tissue of LLS infected ICGV 86699 pool 1	1,6
17	LS2_699	Leaf tissue of LLS infected ICGV 86699 pool 2	1,6
18	Ru1_699	Leaf tissue of Rust infected ICGV 86699 pool 1	1,6
19	Ru2_699	Leaf tissue of Rust infected ICGV 86699 pool 2	1,6
20	SS1_699	Leaf tissue of Salt stressed ICGV 86699 pool 1	1,7
21	SS2_699	Leaf tissue of Salt stressed ICGV 86699 pool 2	1,7
22	DS1_699	Leaf tissue of Drought stressed ICGV 86699 pool 1	1,7
23	DS2_699	Leaf tissue of Drought stressed ICGV 86699 pool2	1,7
24	JL24	Leaf tissue of JL24 peanut variety	1,2,4,5,8
25	TAG24	Leaf tissue of TAG24 peanut variety	1,8
26	CS39	Leaf tissue of CS39 peanut variety	1,8
27	ICGV 00350	Leaf tissue of ICGV 00350 peanut variety	1,8
28	ICGV 05155	Leaf tissue of ICGV 05155 peanut variety	1,8
29	ICGV 06040	Leaf tissue of ICGV 06040 peanut variety	1,8
30	ICGV 86699	Leaf tissue of ICGV 86699 peanut variety	1,2,4,5,8
31	ICGV 91114	Leaf tissue of ICGV 91114peanut variety	1,8

Expression stability of the candidate reference genes was evaluated using two different methods, including geNorm and NormFinder. Firstly, the expression stability of each reference gene and the best combination of normalizer genes for each set of samples were obtained using a pair-wise method by geNorm [20] which is based on the fact that expression ratio of two ideal control genes is identical in all samples and the variation of the expression ratios of two real housekeeping genes reflecting the fact that one (or both) are not constantly expressed, with increasing variation in the ratio corresponding to decreasing expression stability [37]. The raw Cq values were converted into relative quantities after correcting the Cq values according to respective PCR efficiencies for each gene using genEX Professional software (MultiD Analyses AB, Sweden) as the requirement of geNorm analysis to calculate gene expression stability (M). To define the optimal number of genes required for normalization, geNorm platform estimates a normalization factor (NFn) by geometric average of the n best reference genes and performs a stepwise analysis (more stable to less stable genes) to calculate the pair-wise variation (Vn/Vn+1) between two sequential normalization factors, NFn and NFn+1, including more genes in each comparison [46]. The pair-wise variation, to define the optimal number of genes required for normalization was carried out using geNorm of qBase plus software (Biogazelle, Belgium). 

The expression stability of the eight candidate reference genes was also determined by a model-based variance estimation application called NormFinder [47] to rank the candidate reference genes expression stability for all samples with no subgroup determination according to their stability under given set of experimental conditions. The Cq values of each set of samples were converted to relative quantities after efficiency correction for each gene and expression stabilities were calculated by using the NormFinder tool of genEX Professional software (MultiD Analyses AB, Sweden).

### Reference genes validation in transgenic plants

Transgenic peanut plants carrying an antisense PBNV nucleoprotein gene (*PBNVnp*) were selected for validation of candidate reference genes under biotic stress. Similarly, for abiotic stress, transgenic peanut plants transformed with *Arabidopsis thaliana* dehydration responsive element binding factor *1A* (*AtDREB1A*) driven by stress inducible promoter *rd29A* [48] were selected to validate the candidate reference genes under drought stress. Specific primers were designed for *PBNVnp* and *AtDREB1A* genes for estimation of relative expression levels in transgenic plants using qPCR assay.

Stress treatments were carried out as mentioned in the stress treatment section. Leaf sample were collected from five PBNV infected transgenic peanut (four resistant and one susceptible) plants and one uninoculated (healthy) wild peanut (UT- untransformed) plant after five days of inoculation and different levels of viral gene expression expected in these plants, based on their resistance levels. Similarly, a total of six leaf samples were collected from drought stress experiment which included two transgenics and one wild plant (UT- untransformed), before (WW-well watered) and after treatment (DS-drought stressed), where expression of *AtDREB1A* gene was expected only in transgenic plants after drought stress. RNA isolation and qPCR analysis were carried out as mentioned in the previous sections. Relative quantification of target genes was estimated by normalizing with different candidate reference genes using qBase plus software. The mean relative expression values of *PBNVnp* of resistante and suscesptible were presented after scaling with values of healthy UT sample. Similarly mean relative expression values for *AtDREB1A* gene were presented after scaling with values of drought stressed UT sample 

The coefficient of variation was calculated for each reference gene by using the following formula: Coefficient of variation in percentage (CV %) = (standard deviation / mean of Cq)*100

## Results

### Selection of candidate reference genes and primer design

**Figure 1:**
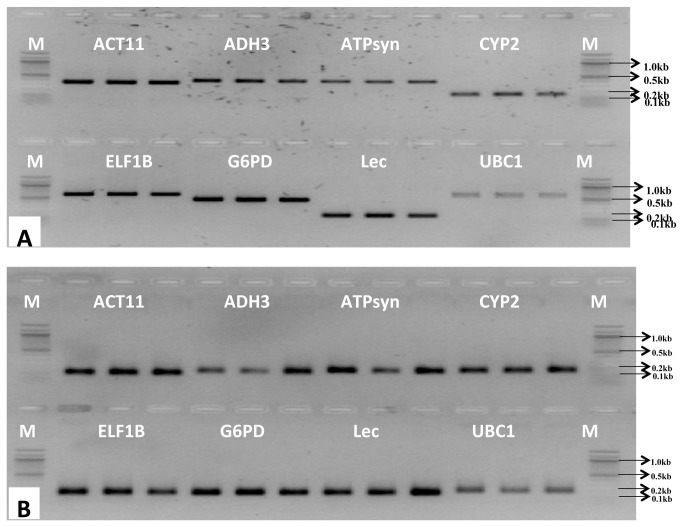
Amplification of a specific PCR product with genomic DNA (A) and cDNA (B) as templates on agarose gel (2.0%) using gene-specific primers for each candidate reference gene. Three replicates of the PCR amplicons with each primer set were loaded; M indicates a100 bp DNA size marker. All primer pairs except *CYP2* and *LEC* amplified a larger size PCR product with DNA template as compared to cDNA template, indicating the position of primer pairs spanning at least one intron.

A real time qPCR assay based on SYBR Green detection was designed for transcript profiling of eight candidate reference genes in 31 diverse samples of peanut. Putative orthologous of six candidate genes including *ACT11*, *ATPsyn*, *ELF1B*, *CYP2*, *G6PD* and *UBC1* were identified from peanut by BLAST searches in the non-redundant and expressed sequence tag (EST) databases at NCBI. Previously reported two peanut gene sequences *ADH3* and *LEC* were also retrieved from NCBI database. Since the genomic sequence of the candidate genes was not available, additional PCRs were carried out with genomic DNA of peanut using gene-specific primers that were used for real-time PCRs to conform the presence of intron(s) within the amplicon region. Most primer pairs except *CYP2* and *LEC* amplified a specific larger-sized PCR product with DNA (Figure 1A) as compared to that with cDNA as template (Figure 1B), thereby indicating that primer pairs for these genes span at least one intron. Since we did not find amplification with total RNA as template with *ELF1B* and *ADH3* primer pairs in PCR, the presence of any genomic DNA contamination in the RNA samples was ruled out. Single expected amplicon and no primer dimer formation with all primer combinations with all samples tested in qPCR indicated specificity and efficiency of the primers (Figure 1 and Figure S1).

### Expression profiling of reference genes

**Figure 2:**
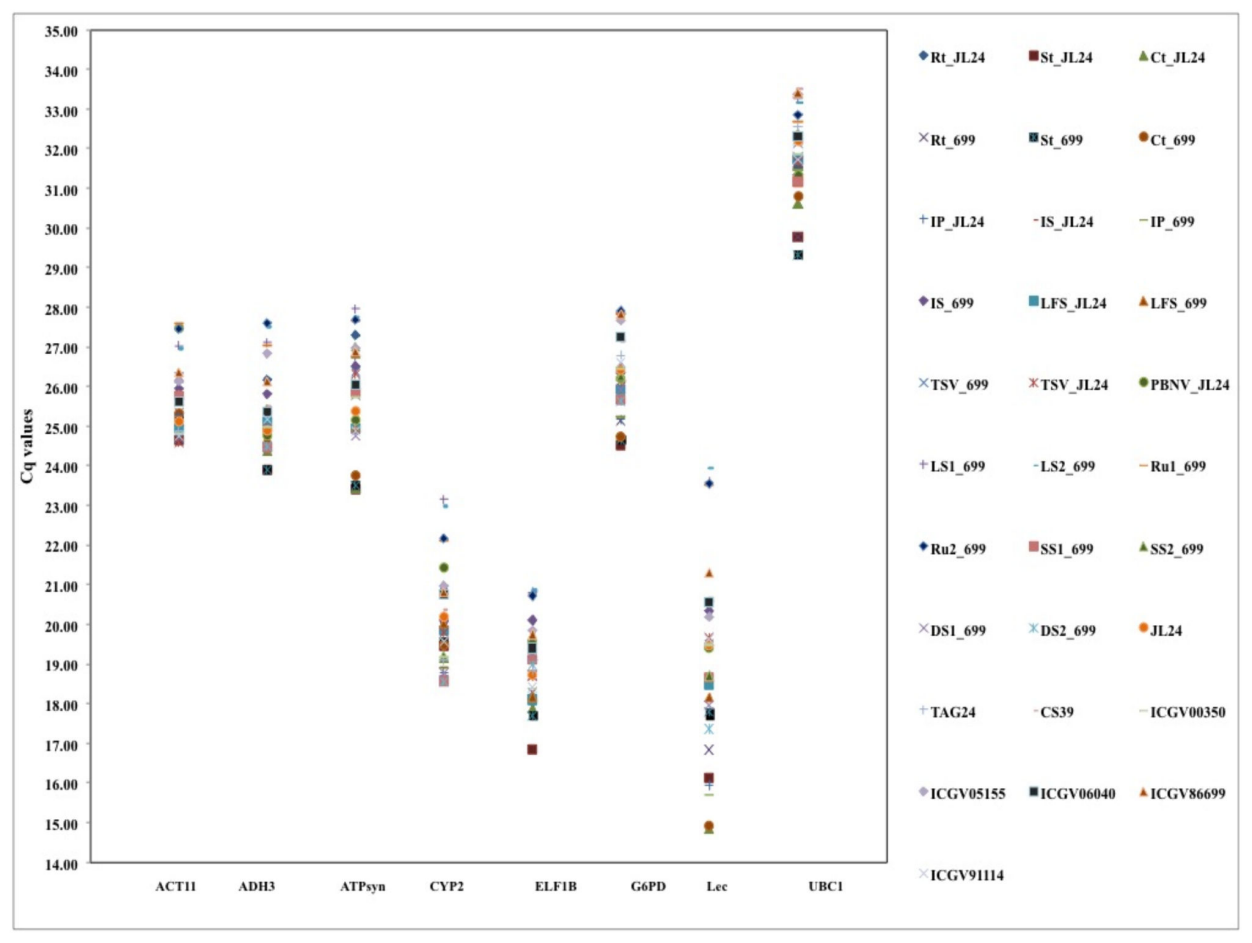
The transcriptional profiles of eight individual candidate reference genes (*ADH3*, *ACT11*, *ATPsyn*, *CYP2*, *ELF1B*, *G6PD*, *LEC* and *UBC1*) in absolute Cq values over all 31 RNA samples tested.

Expression levels of the eight candidate reference genes were determined and assessed for expression stability in a set of 31 diverse tissue samples including eight samples of different peanut varieties, 12 representing vegetative and reproductive stages, and 11 samples from stressed sets representing various biotic and abiotic stress treatments. The amplification plots for each gene were generated and quantification cycle (Cq) was determined for all the tissue samples (Figure 2). The transcript levels of *ELF1B* and *LEC* were higher by several orders of magnitude as indicated by lower average Cq values of 19.09 and 19.25, respectively, than that of other six genes that had average Cq values in the range of 20.15 to 31.82. Among these six genes, *CYP2* was expressed at relatively higher level (average Cq value 20.10) followed by *ADH3* (average Cq value 25.32), *ACT11* (average Cq value 25.74), *ATPsyn* (average Cq value 25.92), *G6PD* (average Cq value 26.26), where *UBC1* exhibited lowest expression with average Cq value of 31.82. The expression levels of eight genes across all the 31 samples ranked as *ELF1B* > LEC > CYP2 > ADH3 > ACT11 > ATPsyn > *G6PD* > *UBC1*.

Individual candidate reference genes had different expression levels across all the sample pools tested. *ACT11* and *G6PD* showed the smallest gene expression variation (below 4 cycles). *ADH3, ATPsyn, CYP2, ELF1B*, and *UBC1* had the expression variation between 4 to 6 cycles, while *LEC* had highest expression variation (above 9 cycles) as shown in Figure 2. The wide expression range of the eight tested candidate reference genes confirmed that no single gene had a constant expression under tested conditions in peanut. Specifically, in the samples set of vegetative stage, the expression levels of the tested genes are highly variable. These results clearly indicate the necessity to select suitable reference genes to normalize gene expression under a certain experimental condition.

### Gene expression stability analysis

**Figure 3:**
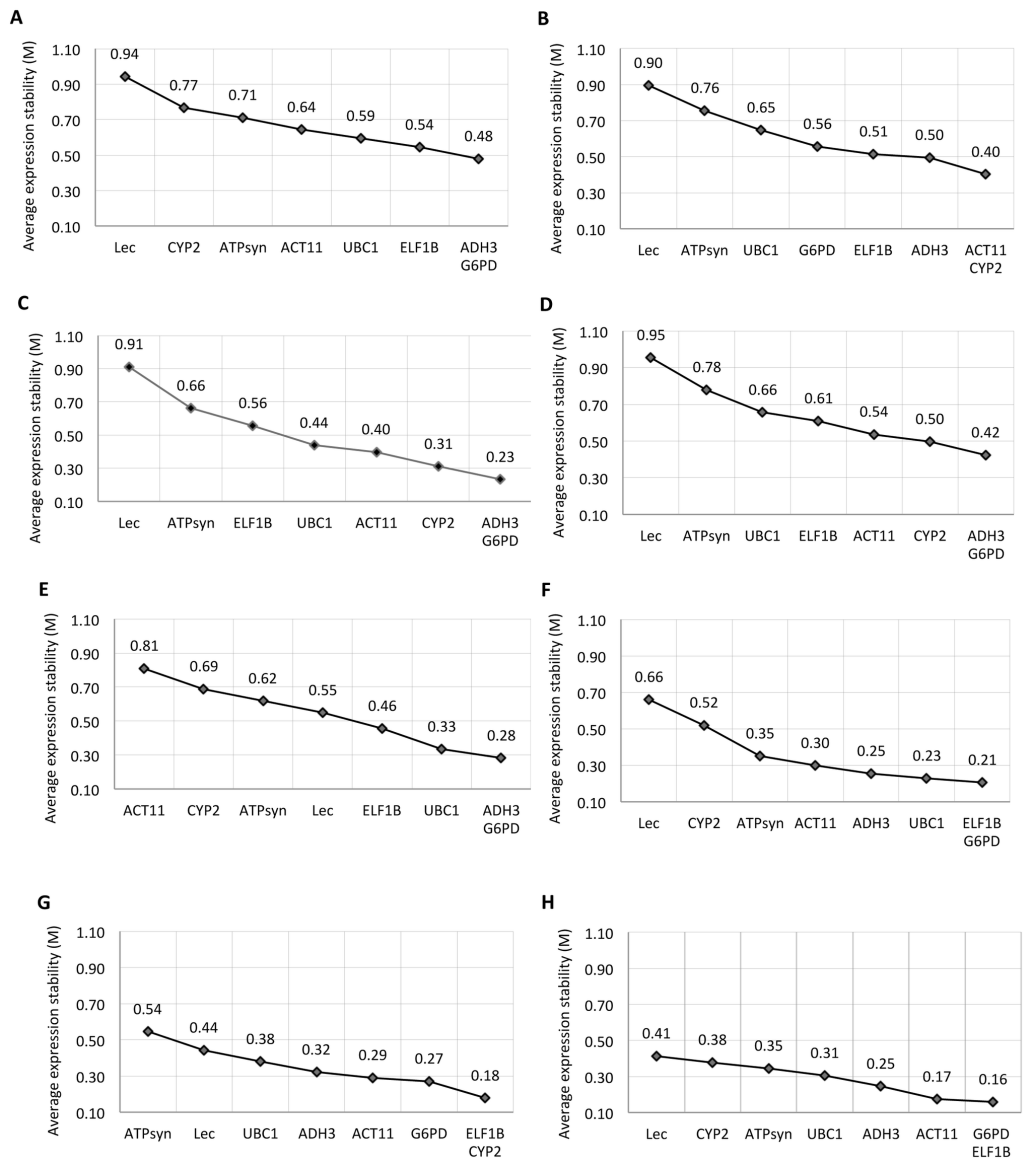
Average expression stability and ranking of eight candidate reference genes using geNorm. All 31 tissue samples set (A), vegetative stage (B), reproductive stage (C), developmental stages (D), viral diseases sample set (E), foliar diseases sample set (F), abiotic stress sample set (G), and different peanut cultivars sample set (H). A lower value of average expression stability (M) indicates more stable expression.

In the entire set of 31 samples, *G6PD* and *ADH3* had lowest (0.48) average expression stability value (M) followed by *ELF1B* (0.54) and *UBC1* (0.59), and M value of *LEC* was highest (0.94) (Figure 3A), thereby suggesting that *G6PD* and *ADH3* had the most stable expression and that *LEC* was expressed most variably. The results remained very similar in the experimental sets of reproductive stage (Figure 3C) and developmental stages (Figure 3D), with the lowest M value for *G6PD* and *ADH3* and M value of *LEC* was highest. In contrast, *CYP2* and *ACT11* were more stable when the vegetative stage samples were analyzed separately, and *LEC* continued as very unstable (Figure 3B). While *G6PD* and *ADH3* gene were most stable under viral infection (TSV & PBNV), *ACT11* was the least stable (Figure 3E). The data set of foliar diseases (LLS-Rust) suggested that *ELF1B* and *G6PD* were most stable, with *LEC* being the least stable (Figure 3F). Under abiotic stress, *ELF1B* and *CYP2* were the most stable genes, while *ATPsyn* was the most variable one (Figure 3G). In the varietal data set, the M value was least for *G6PD* and *ELF1B* followed by *ACT11, ADH3*, *UBC1*, *ATPsyn*, *CYP2*, while *LEC* was again the least stable reference gene (Figure 3H). Notably, the M values for *G6PD* and *ElF1B* in the peanut varietal set were lower (0.16) than those of all other experimental sets, thereby indicating their high expression stability (Figure 3A-H).

The expression stability rankings of candidate reference gene using NormFinder revealed similar results as of geNorm analysis, where the *G6PD* was most stable and *LEC* was least stable genes in the entire sample set, including the reproductive, developmental, and abiotic stress stage sets (Table 3). The stability ranks of the candidate reference genes changed with method (geNorm or NormFinder) used for analysis in the experimental sets of vegetative stage, viral diseases, foliar diseases and in different cultivars sample set. The NormFinder analysis indicated *LEC* gene to be the least stable in all experimental sets except viral diseases, abiotic stress and peanut cultivars. The NormFinder analysis also indicated *ATPsyn* to be the least stable in experimental set of abiotic stress and peanut cultivars, while *ACT11* to be the one least stable under viral disease set (Table 3).

**Table 3:** Gene expression Stability Ranks of 8 candidate reference genes in different sets of peanut samples calculated using geNorm (GN) and NormFinder (NF) methods.

**Sets**	**All samples**		**Vegetative stage**		**Reproductive stage**		**Developmental stages**		**Viral diseases**		**Foliar diseases**		**Abiotic stress**		**Peanut cultivars**	
**Gene**	**GN**	**NF**	**GN**	**NF**	**GN**	**NF**	**GN**	**NF**	**GN**	**NF**	**GN**	**NF**	**GN**	**NF**	**GN**	**NF**
*G6PD*	1	1	5	1	1	1	1	1	1	2	1	6	3	3	2	3
*ADH3*	2	5	3	2	2	3	2	2	2	3	4	2	5	5	4	5
*ELF1B*	3	6	4	3	6	6	5	3	4	1	2	4	2	2	1	1
*UBC1*	4	4	6	7	5	5	6	7	3	7	3	5	6	6	5	4
*ACT11*	5	7	2	5	4	4	4	5	8	8	5	3	4	4	3	2
*ATPsyn*	6	2	7	6	7	7	7	6	6	6	6	1	8	8	6	8
*CYP2*	7	3	1	4	3	2	3	4	7	4	7	7	1	1	7	7
*LEC*	8	8	8	8	8	8	8	8	5	5	8	8	7	7	8	6

### Optimal number of internal candidate genes for normalization

**Figure 4:**
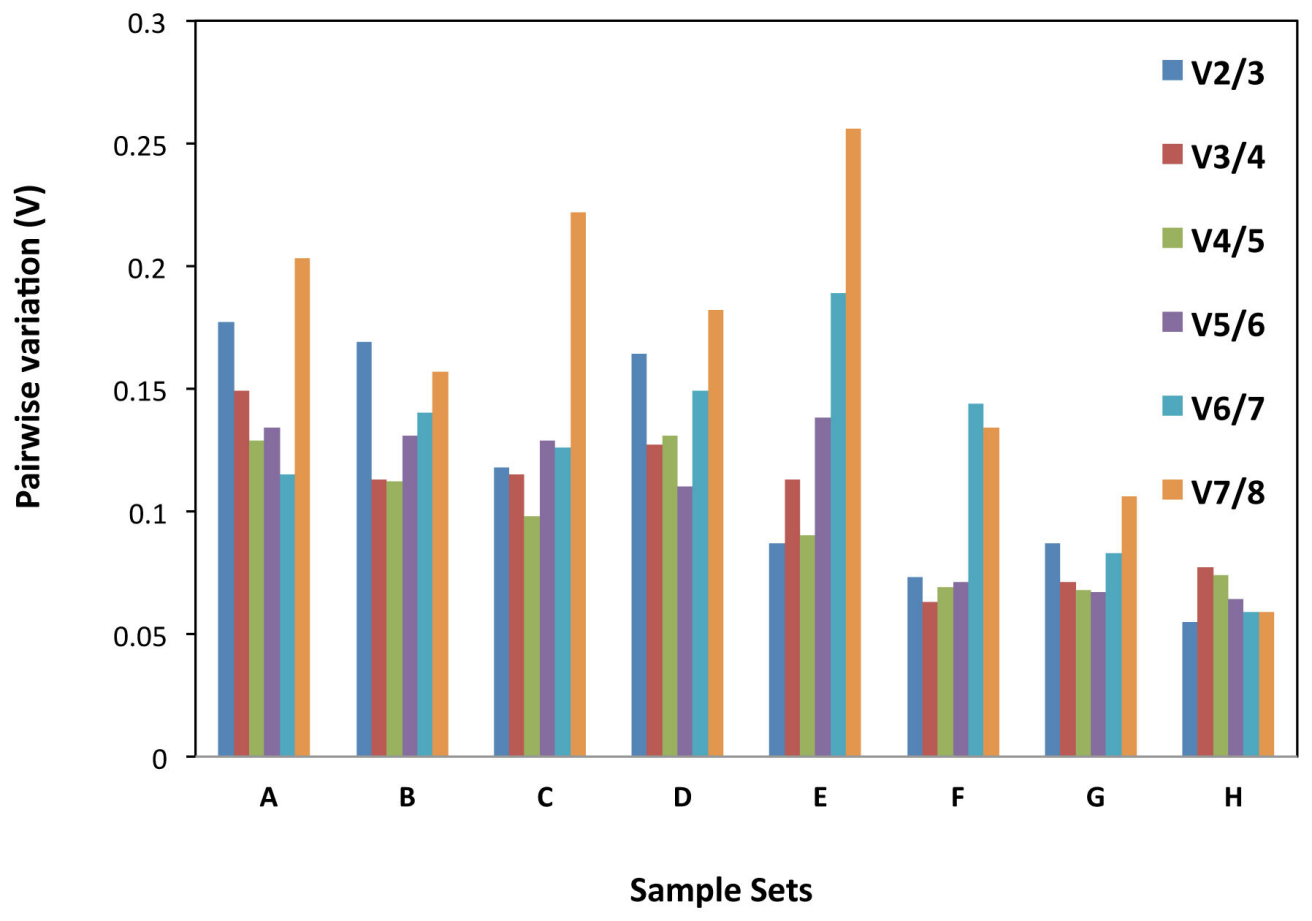
Determination of the optimal number of reference genes for normalization by pair-wise variation using geNorm. All 31 tissue samples set (A), vegetative stage (B), reproductive stage (C), developmental stages (D), viral diseases sample set (E), foliar diseases sample set (F), abiotic stress sample set (G) and different peanut cultivars sample set (H). The pairwise variation (Vn/Vn+1) was analyzed between normalization factors NFn and NFn+1 by geNORM program to determine (V<0.15) the optimal number of reference genes.

We used geNorm to determine the pairwise variation in eight experimental sets of the samples. When all 31 samples were taken together, the pairwise variation V2/3 was higher than 0.15 (0.176) whereas V3/4 was 0.149 (Figure 4), indicating that *ADH3* and *G6PD* genes together are not sufficient for normalization, and hence need a third gene *ELF1B*. Similarly the vegetative stage and developmental stages sets too required three genes for normalization viz., *CYP2, ACT11* and *ADH3* for vegetative stage*, ADH3*, *G6PD* and *CYP2* for developmental stages, as indicated by their pairwise variation value V2/3 were higher than 0.15 and V3/4 were less than 0.150 (Figure 4).

In the other five experimental sets, addition of the third reference gene for normalization of gene expression showed no significant effect as indicated in pairwise variation (Figure 4). However, different experimental sets required a different pair of genes for normalization of gene expression, as indicated by pairwise variation analysis of *G6PD* and *ADH3* genes for sets of reproductive stage and viral diseases, *ELF1B* and *G6PD* genes for foliar diseases (LLS and Rust), and the varietal set, whereas *CYP2* and *ELF1B* genes for abiotic stress (drought and salinity) were sufficient for normalization of gene expression in peanut. When evaluating all the pairwise variations, the least stable reference gene was found to be *LEC* followed by *ATPsyn* (Figure 4).

### Reference genes validation

**Figure 5:**
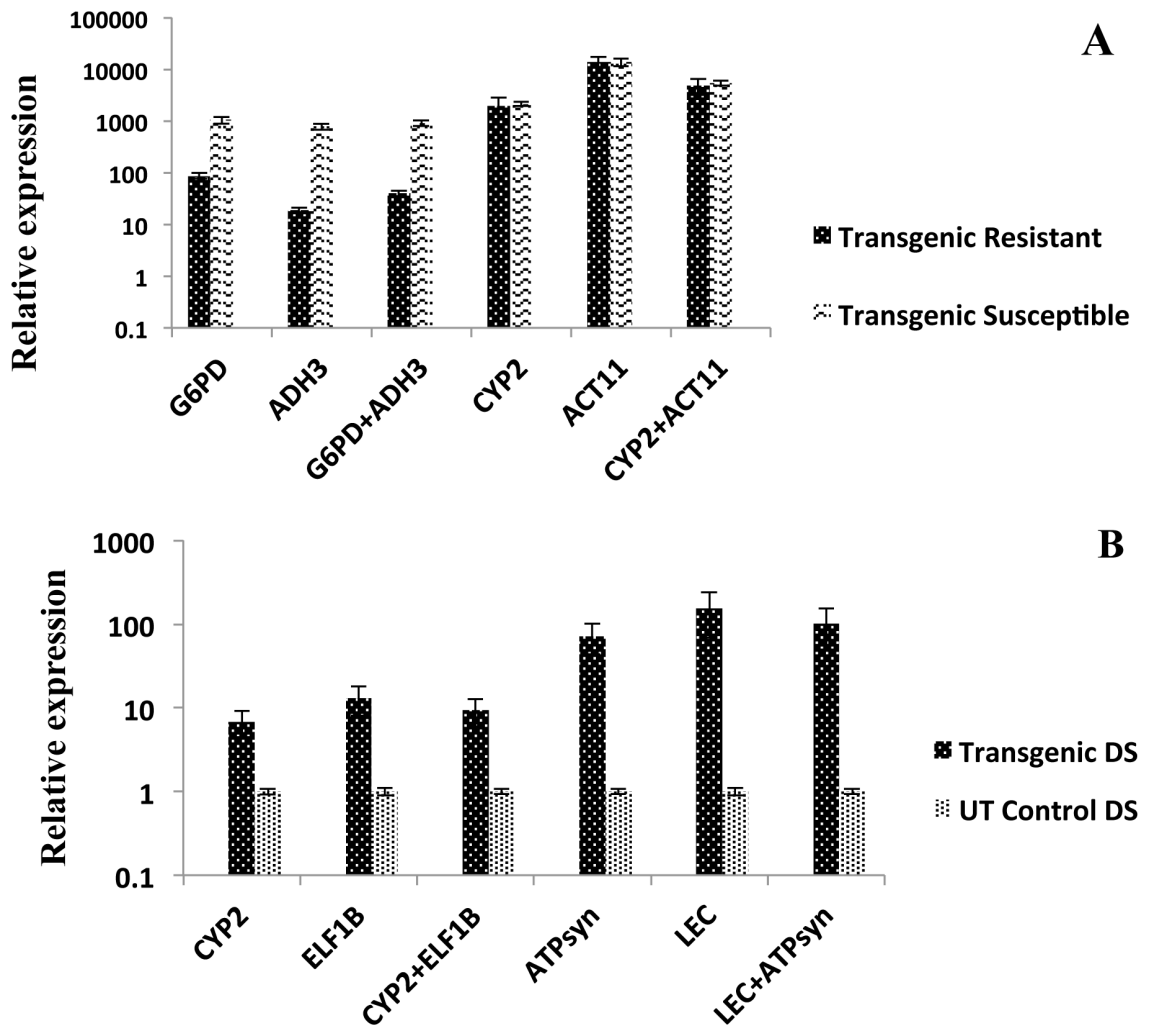
Relative quantification of *PBNVnp* and *AtDREB1A* genes to validate candidate reference genes of peanut under biotic and abiotic stress conditions. (A) Expression of *PBNVnp* gene in infected transgenic peanut leaf sample relatively quantified with candidate reference genes. (B) *AtDREB1A* gene expression in leaf sample of transgenic (rd29a:AtDREB1A) peanut relatively quantified with candidate reference genes. The relative quantity values were presented after scaling to control samples in both the (*PBNVnp* and *AtDREB1A*) cases.

Transgenic peanut samples from biotic and abiotic stress treatments were used for validation of candidate reference genes. PBNV infected transgenic peanut plants carrying antisense nucleoprotein gene of PBNV (*PBNVnp*) were used for validation under biotic stress. The relative expression levels of the target *PBNVnp* gene were presented after scaling with uninoculated UT control in each normalization analysis. The relative expression levels of *PBNVnp* gene in infected peanut samples were similar when normalized with candidate reference genes *G6PD* or *ADH3* individually and in combination, while normalization with *CYP2* or *ACT11* genes showed a different pattern and a very high relative expression of *PBNVnp* gene in both the infected samples including resistant and susceptible (Figure 5A). The results were in accordance with the obtained phenotypic data (data not included), when normalized with *G6PD* and *ADH3* reference genes, whereas normalization with *CYP2* or ACT11 did not correlate with the phenotypic data. 

Similarly, the *AtDREB1A* gene expression levels in transgenic peanut under drought stress were estimated by normalizing with the candidate reference genes. The expression levels of the target gene *AtDREB1A* were presented after scaling with drought stressed UT sample in each normalization analysis. The *AtDREB1A* gene expression levels increased several fold when normalized with *ATPsyn* and *LEC* individually or in combination, compared with the values obtained after normalization with *CYP2* and *ELF1B* genes (Figure 5B).

The percentage of coefficient of variation (CV %) calculated for each reference gene in *PBNVnp* gene expression validation studies indicated high CV with *CYP2* (13.8 %) and *ACT11* (17.2 %) genes, while *G6PD* (3.7 %) and *ADH3* (5.4 %) showed a lower CV. Similarly, the CV was lower for *CYP2* (2.7 %) and *ELF1B* (4.7 %) reference genes and higher for *ATPsyn* (11.7 %) and *LEC* (14.0 %) genes under *AtDREB1A* gene expression studies. This data is in accordance with stability rankings of the candidate reference gene under biotic and abiotic stress conditions.

## Discussion

Real-time PCR (qPCR) is a very powerful technique to quantify the expression levels of target genes and stably expressing reference genes required for data normalization to minimize the experimental errors in relative quantification. Nevertheless, no genes are stably expressed universally in any organism, and are regulated to only a certain extent [49]. Several studies on reference gene validation have insisted that multiple internal genes must be evaluated in order to improve the accuracy of a qPCR analysis and interpretation of gene expression [20,50,51]. The present study describes a comprehensive analysis on the validation of eight candidate reference genes in 31 diverse samples of peanut, divided broadly into eight experimental sets. Our analysis based on geNorm and NormFinder algorithms indicate that the choice of reference genes for normalization should be experimental condition-specific. In the present study, we tested the expression stability of commonly used housekeeping genes like *ACT11*, *ELF1B*, *CYP2*, and *ADH3* that have been previously described as the most stable genes in various plant studies. Some of these housekeeping genes like *ADH3* [37] and *ACT* [38] that qualify among the best reference genes under specific experimental sets of peanut were not found to be suitable reference gene across all the eight experimental sets. 

The average expression levels of *ELF1B* and *LEC* genes were higher by several orders of magnitude than that of other six genes. The expression levels of *LEC* gene were very high in immature seeds and pods compared to other samples and unstable among the tested samples, whereas the *ELF1B* gene expression is high and stable in all the samples tested. The average expression level of *UBC1* gene was lowest among the eight genes tested across experimental sets. This might be due to a possible single copy presence of Ubiquitin-conjugating enzyme (*UBC1*) gene in the tetraploid genome of peanut as previously reported in cotton [44].

Gene expression stability (M) of eight candidate reference genes in various sets of tissue samples of peanut under different experimental conditions was measured by geNorm which calculates the mean pairwise variation for a gene in comparison to all other genes being tested and reports the average expression stability (M) of all the genes in a given set of samples [20]. Genes with the lowest M value have the most stable expression, while the highest M value indicates the least stable expression. In the present study, the candidate reference genes displayed high expression stability in all eight experimental data sets indicated by low (<1.0) M values. The *G6PD* (glucose-6-phosphate 1-dehydrogenase) gene was most stable among the eight genes tested across the six experimental sets, except during the vegetative stage and abiotic stress sets. This is in contrast to the previous report in soybean where *G6PD* gene expression was least stable under different photoperiodic treatments and developmental stages [29] and under cadmium stress [33]. The *G6PD* gene has been involved in the glycolytic pathway similar to GAPDH (glyceraldehyde-3-phosphate dehydrogenase) that has been the most commonly reported reference gene [17,38]. Unlike *G6PD*, *GAPDH* was recently reported as stably expressing reference gene in two legumes including chickpea [17] and peanut [38]. These results indicate that stability of gene expression is purely based on experimental condition and not only on the species tested.

The second most stably expressing gene in this study was *ADH3* that was most stable in the entire sample set including the reproductive stage, developmental stages and in viral diseases experimental sets. The *ADH3* encoding for alcohol dehydrogenase class III enzyme that catalyzes the inter-conversion of alcohols and aldehydes or ketones with the reduction of NAD^+^ to NADH plays an important role in lowering the toxicity of the cell [52]. Although the *ADH3* has not been used frequently as a qPCR reference gene, previous reports showed its stable expression in *coffea arabica* [53] and peanut [37]. While in the previous study with peanut, *ADH3* was restricted to kernel developmental stages [37], our study included different experimental sets from developmental stages, biotic stress, abiotic stress and a range of peanut varieties.

The *ELF1B* (Elongation factor 1-beta) and *CYP2* (Cyclophilin) genes were found to be next in ranking in terms of their stable expression after *G6PD* and *ADH3*. While the *ELF1B* was most stable under foliar diseases and across peanut varieties, *CYP2* was most stable in vegetative stages, and both *ELF1B* and *CYP2* were most stable under abiotic stress. These observations are in accordance with other studies where *ELF1B* gene under cold stress in peanut [39] and under abiotic stress [32] and cadmium stress [33] in soybean was reported as most stably expressed. Similarly, *CYP2* was reported as most stably expressed gene in different experimental sets of *Vicia faba* [54]. *ELF1B* and *CYP2* also showed stable expression in different tissues under various developmental stages of soybean [29]. *ACT1*, a member of Actin gene family has so far been considered to be the most stable across different species of peanut [38], whereas *ACT11* has been considered to be the most stable in all the samples tested in peanut [39]. Nevertheless, in the present study *ACT11* gene was found to be stable only during the vegetative stages of peanut, indicating that its expression might have been influenced by the experimental conditions in this study.

Our data clearly demonstrates the unsuitability of *LEC* (lectin) gene as a reference gene for gene expression studies in peanut where it showed the least stable expression across different experimental sets analyzed. Similarly, *ATPsyn* (ATP synthase) gene that is involved in the synthesis of adenosine triphosphate (ATP) has so far not been reported as stably expressing internal gene, with only one report where it was used as an endogenous gene in soybean [43]. Since *ATPsyn* was not stable across the experimental conditions, it is not recommended as a reference gene for gene expression studies in peanut.

Although, a single reference gene with high expression stability may be appropriate for normalization of gene expression data in some experimental conditions, in most of the experimental conditions, there may be no single gene suitable as a reliable reference gene and two or more internal reference genes are required for accurate and reliable results [20]. The pairwise variation results in our study indicated that the different pairs of most stable reference genes were found to be optimal for the accurate normalization across the five experimental sets, viz., reproductive stage, viral diseases, foliar diseases, abiotic stresses and different peanut varieties, where pairwise variation values (V2/3) were lower than the cut-off value of 0.15 [20]. Addition of the third candidate reference gene was necessary only to normalize gene expression in sets of all samples, developmental stages and vegetative stage. The results of these three sets indicated unstable expression of genes in the vegetative stage samples. 

Candidate reference genes of peanut were validated with transgenic peanut plants under biotic and abiotic stresses with *PBNVnp* and *AtDREB1A* genes, respectively. Normalization of *PBNVnp* gene expression in infected transgenic peanut plants showed variable expression levels with *CYP2* and *ACT11* genes as compared to those obtained by normalizing with *G6PD* and *ADH3* reference genes indicating lower stability of *CYP2* and *ACT11* genes. Similarly, normalization of stress inducible *AtDREB1A* gene expression in transgenic peanut with *CYP2* and *ELF1B* reference genes indicated its stability under abiotic stress when compared to *LEC* and *ATPsyn* genes that had shown extreme expression levels, indicating their instability under the tested experimental conditions. These validation results indicated that stability ranks of the tested candidate reference genes are accurate and more than one stably expressed reference gene should improve the accuracy of normalization.

## Conclusion

In the present study, we validated eight candidate reference genes by extending the study to diverse samples including a range of biotic and abiotic stresses, different developmental stages and cultivars. While the *ADH3* and *G6PD* exhibited the most stable expression in all the tissue samples, the *ELF1B* expression was stable across different varieties, foliar diseases and abiotic stress conditions. Although, two genes like *ADH3* and *G6PD* should be sufficient to give reliable results, the addition of a third gene *ELF1B* as reference gene may produce even better results in a diverse set of tissue samples of peanut.

## Supporting Information

Figure S1 **Melting curves of the 8 candidate reference genes of peanut: A-*ACT11*, B-*ADH3*, C-*ATPsyn*, D-CYP2, E-*ELF1B*, F-*G6PD*, G-LEC, H-*UBC1*.**
(TIF)
