# Supplementary figures and images for: Evaluation and Validation of Reference Genes for Normalization of Quantitative Real-Time PCR Based Gene Expression Studies in Peanut
Source: PLoS One. 2013 Oct 22;8(10):e78555. doi: 10.1371/journal.pone.0078555 (PMC3805511; doi:10.1371/journal.pone.0078555)

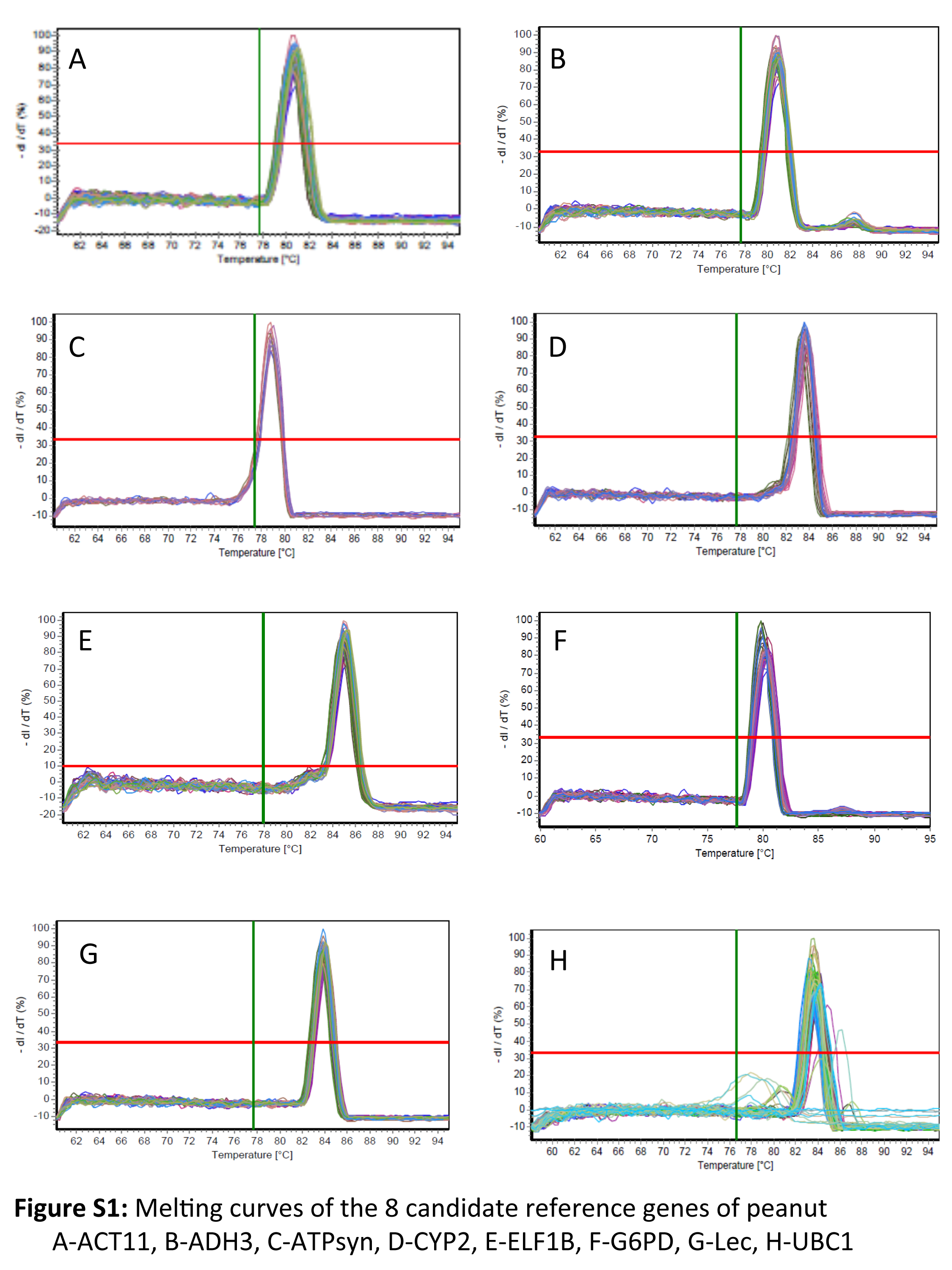

Supplement: Figure S1 — Melting curves of the 8 candidate reference genes of peanut: A-ACT11, B-ADH3, C-ATPsyn, D-CYP2, E-ELF1B, F-G6PD, G-LEC, H-UBC1. (TIF) [file pone.0078555.s001.tif]
